# Patterns of avian haemosporidian infections vary with time, but not habitat, in a fragmented Neotropical landscape

**DOI:** 10.1371/journal.pone.0206493

**Published:** 2018-10-31

**Authors:** Juan Rivero de Aguilar, Fernando Castillo, Andrea Moreno, Nicolás Peñafiel, Luke Browne, Scott T. Walter, Jordan Karubian, Elisa Bonaccorso

**Affiliations:** 1 Centro de Investigación en Biodiversidad y Cambio Climático, Universidad Tecnológica Indoamérica, Quito, Pichincha, Ecuador; 2 Fundación para la Conservación de los Andes Tropicales, Quito, Pichincha, Ecuador; 3 Department of Ecology and Evolutionary Biology, Tulane University, New Orleans, Louisiana, United States of America; 4 Department of Biology, Texas State University, San Marcos, Texas, United States of America; 5 Instituto BIOSFERA y Colegio de Ciencias Biológicas y Ambientales, Universidad San Francisco de Quito, Quito, Pichincha, Ecuador; Instituto Rene Rachou, BRAZIL

## Abstract

Habitat loss has the potential to alter vertebrate host populations and their interactions with parasites. Theory predicts a decrease in parasite diversity due to the loss of hosts in such contexts. However, habitat loss could also increase parasite infections as a result of the arrival of new parasites or by decreasing host immune defenses. We investigated the effect of habitat loss and other habitat characteristics on avian haemosporidian infections in a community of birds within a fragmented landscape in northwest Ecuador. We estimated *Plasmodium* and *Haemoproteus* parasite infections in 504 individual birds belonging to 8 families and 18 species. We found differences in infection status among bird species, but no relationship between forest fragment characteristics and infection status was observed. We also found a temporal effect, with birds at the end of the five-month study (which ran from the end of the rainy season thru the dry season), being less infected by *Plasmodium* parasites than individuals sampled at the beginning. Moreover, we found a positive relationship between forest area and *Culicoides* abundance. Taken as a whole, these findings indicate little effect of fragment characteristics per se on infection, although additional sampling or higher infection rates would have offered more power to detect potential relationships.

## Introduction

Habitat loss caused by human activity is a primary cause of biodiversity loss worldwide [1,2]. This process is particularly pronounced in the Neotropics, where much of Earth’s biodiversity is located [3]. One of the main effects of habitat loss on biodiversity is the alteration of biological interactions, including the relationships between host and parasites [4,5]. Whereas the effect of habitat loss on vertebrate hosts is generally well studied [6–8], its effect on parasite loads is less understood [9]. This is in part because of the complexity of concurrently studying hosts, vectors, and parasites involved in host-parasite interactions in natural populations. Undisturbed habitats, given their larger size and potentially increased niche diversity, are expected to harbor a higher diversity of hosts and vectors compared to disturbed areas, therefore yielding a more diverse community of parasites [10–12]. Corroborating these expectations, high prevalence or intensity of infection and high parasite diversity have been observed in undisturbed habitats in vertebrates, including birds [10,13–15]. However, a reduced prevalence of infection has been also observed in undisturbed habitats compared to disturbed ones, perhaps because of a dilution effect related to an elevated host diversity [16–18]. On the other hand, habitat loss is sometimes associated with low parasite infections, mainly explained by reduction on host and vectors abundance [19]. Moreover, disturbed habitats have been observed to harbor high parasite richness and abundance, in this case due to changes in environmental conditions [20], arrival of new host species, parasites, and vectors [21–23] or overcrowding of hosts [21,24]. These complex and contradictory results highlight the need for more research from natural systems, especially in tropical areas where, in addition to high deforestation rates, species interactions are generally poorly known.

The avian haemosporidian system is well suited to advance our understanding how habitat loss may influence host-parasite interactions. This group is composed of protozoans of the genera *Plasmodium*, *Haemoproteus*, *Leucocytozoon* and *Fallisia* [25,26]. These parasites have a global distribution and commonly infect birds in the wild. They are commonly found as mild chronical infections but also produce detrimental effects on fitness and survival [27,28]. They depend on dipteran vectors for their transmission [29], which in turn are influenced by environmental conditions and habitat quality [30]. There is evidence that host abundance and diversity may affect the parasite diversity and specialization [31]. For example, *Plasmodium* parasites behave more generalist than *Haemoproteus* parasites, yet in areas where host diversity is potentially high, as in the Neotropics, both parasite genera could behave as generalists [31]. Morevover, parasites’ transmission depends on vector ecology and activity[32,33]. Therefore, complementary knowledge of parasite specificity and transmission in different ecological scenarios is necessary to disentangle host-parasite interactions.

To investigate associations between habitat loss and parasite infections, we evaluated haemosporidian parasites infecting birds in a fragmented landscape in northwest Ecuador. In the study area, a continuous forest has been converted to small fragments during the last 30–50 years [34], providing a framework to explore host-parasite interactions in a landscape characterized by habitat loss. We captured birds from different forest fragments to assess the degree to which patterns of haemosporidians infection were explained by forest characteristics. Given that many of the fragments in our study system have been isolated for several decades, and bird and vector diversity depend on forest structure/quality, we expected to find a reduction of parasite infections in the most degraded or isolated forest fragments. We also evaluated a potential effect of seasonality on haemosporidian infections because the study which ran from the end of the rainy season thru the dry season. Precipitation has been observed to increase prevalence and parasitemia of haemosporidians in tropical forests [35]. In this case, we predicted that individuals sampled at the end of the rainy season should have more infections, compared to the dry season, because of a higher abundance and activity of mosquitoes. To test these predictions, we specifically: 1) estimated bird infection status (presence/absence of infection) of *Plasmodium* and *Haemoproteus* parasites across avian species across fragments; 2) investigated the phylogenetic relationships among parasites lineages to better understand the specialist/generalist relationship of parasites; 3) estimated haemosporidians-borne mosquito abundance in fragments; 4) measured seasonality and variables associated with forest quality and habitat; and, 5) investigated the effect of forest characteristics on mosquitoes and haemosporidians infections.

## Methods

### Study area

This study was conducted from 1 August to 20 December, 2014, which ran from the end of the rainy season thru the dry season, in the Mache-Chindul Ecological Reserve (REMACH), Esmeraldas province, Ecuador (79^o^ 48.00' W, 0^o^ 24.60' N). The reserve covers an altitudinal range of 200–800 m a.s.l. and is located in the Chocó biographic region. REMACH is characterized by high humidity and high precipitation (2000 mm annual mean with a dry season from July to December [36]), and has an area of 119,172 ha consisting of fragments of primary and secondary forest surrounded by a matrix of agricultural lands. The center of REMACH is further protected by Bilsa Biological Station (BBS, 3500 ha), the main continuous forest within the reserve. Our study focused on forest fragments, usually found on steep hills, where human intervention is limited. REMACH is one of the most diverse areas of Ecuador in terms of bird species, with at least 360 species recorded to date, many of which are threatened or endemic to Ecuador [36,37].

### Bird sampling

**Fig 1:**
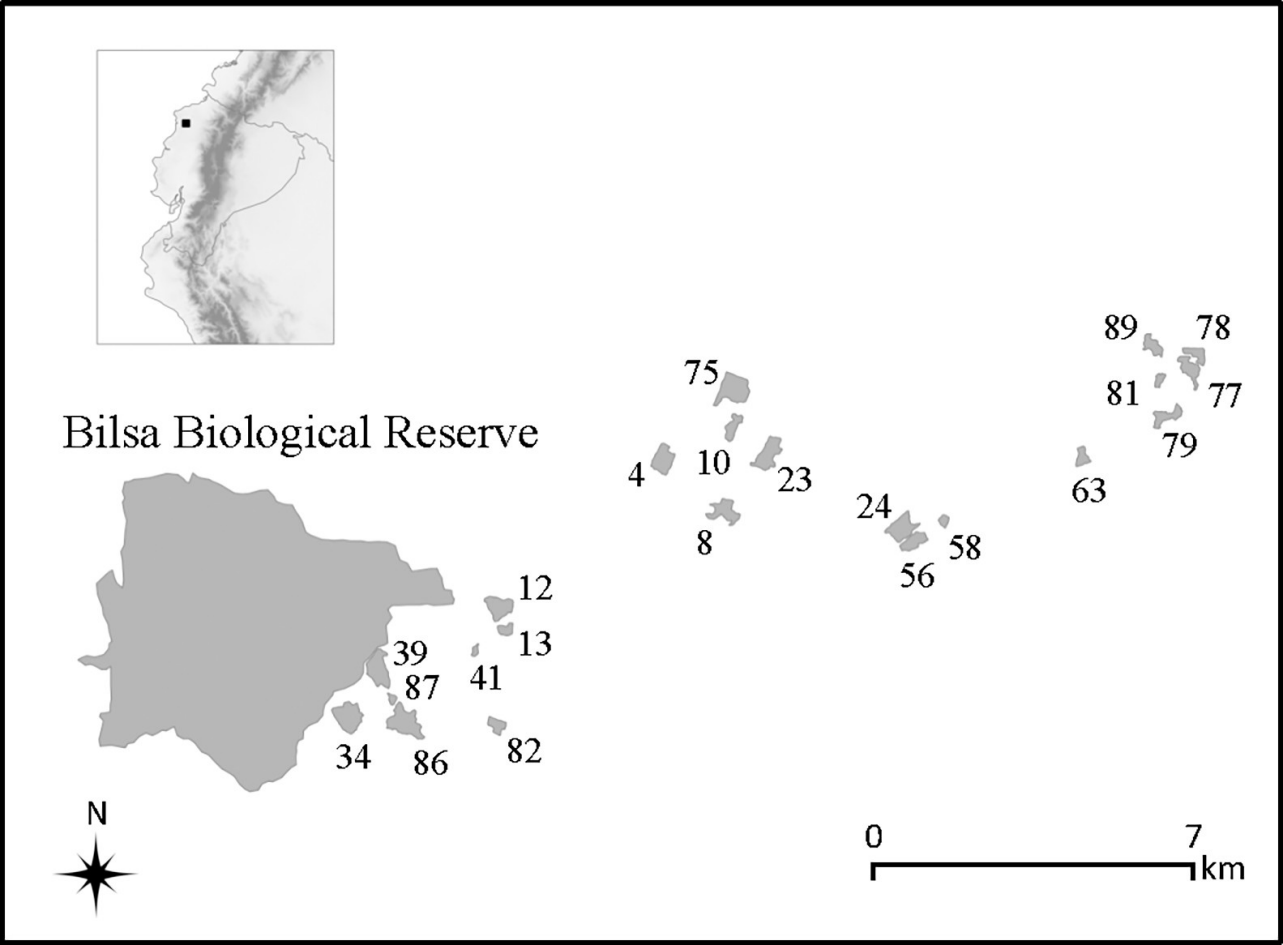
Map of sampling sites in the Mache-Chindul Ecological Reserve (REMACH), Esmeraldas province, northwest Ecuador. Numbers represent forest fragments sites.

Bird sampling was performed in 22 forest fragments situated within REMACH (Table 1 and Fig 1) around five small towns. Each month we carried out mist-netting for 20 consecutive days in fragments around each town. Birds were captured by using eight mist-nets (12 m, 36 × 36 mm mesh) in each fragment for 3 consecutive days, with four nets placed near the forest edge and four placed inside the forest, approximately 300 m from the edge. The first two days, mist-nets were active from 6:30 to 13:30; the third day they were open from 6:30 to 12:00 to allow moving to the next fragment to set-up the mist-nets. Bird species were identified following Ridgely and Greenfield [38]) and banded with standard aluminum metal rings. We followed the nomenclature of the South American Classification Committee (SACC) [39]. Bird sampling was approved by the Tulane University Animal Care Committee (IACUC- 395). The perimeter of each forest fragment was mapped by walking the boundaries using a GPS unit, and a 500 m transect was established from the edge to the center of each fragment.

**Table 1:** Forest fragments sampled in Mache-Chindul Ecological Reserve (REMACH) northwest Ecuador.

Fragment	Longitude	Latitude	Area	Cover	PCA	Elev	Date	*Culicoides*	*Culex*	Shannon
4	-79.64816609	0.381915724	21.3	91.4	1.5	374.4	1	-	-	0.3
8	-79.62884996	0.371602802	20.5	91.6	-1.1	232.7	20	111	20	0.5
10	-79.634374	0.388416685	11.6	88.4	-0.1	374.6	7	-	-	-1.1
12	-79.68052353	0.352561058	21.4	91.1	0.9	518.8	71	122	0	0.5
13	-79.67921242	0.34806556	6.7	83.8	0.6	537.5	60	26	2	0.6
23	-79.62740299	0.38298873	24.8	93.5	2.6	364.4	17	230	0	-1.3
24	-79.60104539	0.368314106	23.3	92.9	0.1	239.3	124	2	2	-0.8
34	-79.71030131	0.330766801	29.7	91.1	1.1	550.1	92	86	1	0.9
39	-79.70434328	0.340227071	27.7	84.4	-2.4	591.8	95	19	0	1.1
41	-79.68524161	0.343869569	2.6	84.6	0.4	546.7	64	45	2	-0.4
56	-79.59864702	0.365591306	14.1	92.7	0.7	236.3	121	17	1	1.5
58	-79.5927168	0.369714693	4.2	83.1	-0.1	217.3	127	19	3	-1.1
63	-79.56543884	0.382408713	8.3	84.8	-1.5	174.7	130	94	3	-0.8
75	-79.63428294	0.395896655	33.6	90.3	1.7	399.5	13	117	1	-0.6
77	-79.54418851	0.399860744	15.5	83.2	-0.2	159.8	45	184	30	-1.6
78	-79.54292124	0.402827069	9.9	83.7	-2.1	147.9	41	31	2	-1.6
79	-79.54874505	0.390527748	13.5	83.5	-1.1	222.9	32	23	0	-0.1
81	-79.55018125	0.397727335	4.5	86.4	0.8	134.5	38	4	0	-1.1
82	-79.68099392	0.329062482	9.2	86.6	0.8	376.6	67	75	0	1.6
86	-79.69913361	0.329661848	33.6	92.2	-0.3	493.7	102	485	0	0.3
87	-79.70162173	0.334302245	3.11	88.4	-1.9	536.1	98	22	0	1.4
89	-79.55152766	0.404528961	11.58	81.2	-0.6	168.6	35	3	3	0.2
		Mean ± SD	15.9±9.8	87.6±3.9	-0.009±1.2	345.3±157.3	63.6±41.9	85.7±113.1	3.65±8.1	-0.07±1.01

*Abbreviations*: *Area* forest fragment area (in ha), *Cover* tree cover around fragments (%), *PCA* habitat type principal components analysis, *Elev* meters above sea level (m a.s.l.), *Date* date of bird sampling, *Culicoides* number of *Culicoides* individuals captured in the light trap, *Culex* number of *Culex* individuals captured in the light trap, *Shannon* Shannon bird diversity, *SD* standard deviation.

### Blood sampling and DNA extraction

For parasite infection diagnosis, we took a blood sample (10–50 μl) from birds from the brachial vein, and from the tarsal vein of hummingbirds. A blood smear was immediately prepared from a blood drop and air dried in the field. Smears were fixed with ethanol 96%, air dried [29], and stained with Giemsa (Panreac, EU) diluted in buffer solution pH 7.2 DC (Panreac, EU), for 1 h. The rest of the blood was stored at room temperature in criotubes with 100% alcohol for posterior parasite molecular identification. Genomic DNA from blood samples was extracted using guanidine thyocianate protein precipitation [40]. We only analyzed bird species with at least 10 individuals captured [33,41].

### Parasite identification

*Plasmodium* and *Haemoproteus* parasites were identified both by light microscopy and by PCR amplification. Blood smears were inspected using light microscopy (Olympus CX31) to visually search for any stage of the parasite. Identification of parasites was based on photo smears of haemosporidian parasites from international databases [42,43] and according to the methods proposed by Valkiunas et al. [29]. Infection status was determined by inspecting 2000 erythrocytes at 1000x magnification under oil immersion, and all smears were inspected by the same person (JRA). If no infection was observed by microscopy, but a positive infection was detected by PCR (a sign of a low parasitemia), the smear was inspected again by observing a total of 10000 erythrocytes. Parasites that were impossible to assign to either genera of parasite by microscopy were assigned to genera by PCR.

Molecular diagnosis by PCR was based on the amplification of a portion of the parasite’s cytochrome b gene (478 nucleotides excluding primers). Every PCR reaction contained MgCl_2_ 2.5 mM, dNTPs 0.4 mM each, primers at 0.6 μM, 1.25 U Taq polymerase (Invitrogen), and 5 μL DNA in a total volume of 25 μL. All individuals that resulted negative in this first PCR were tested again, performing a second PCR, using a 1:50 dilution of the product of the first one as template. The second PCR was set up as follows: MgCl_2_ 3 mM, dNTPs 0.2 mM each, primers at 0.2 μM, 1.25 U Taq polymerase (Invitrogen), and 1 μL of template in a total volume of 25 μL. Amplification was conducted using primers HAEMF (5'-ATGGTGCTTTCGATATATGCATG-3') and HAEMR2 (5'-GCATTATCTGGATGTGATAATGGT-3') [44], which are suited to detect both parasites simultaneously.

DNA from an infected individual was used as a positive control, and purified water in place of DNA template was used as a negative control. Temperature profile for the first PCR consisted of 3 minutes at 94°C, followed by 35 cycles of 94°C for 30 seconds, 56°C for 30 seconds, and 72°C for 45 seconds; with a final extension step of 10 minutes at 72°C. The second PCR followed the same structure, but only 20 cycles were performed instead of 35. PCR products were visualized in 1.5% agarose gel, and amplicons (positive diagnosis) were purified with illustra ExoProStar (GE Healthcare Life Sciences) following manufacturer’s instructions. Sequencing of both DNA strands was performed by Macrogen Inc. (Seoul, Korea), using PCR primers and BigDye chemistry, and screening in ABI3730XL. Resulting DNA chromatograms were inspected to evaluate their quality and search for double peaks (a signal of mixed infections). If double peaks were observed and to resolve the presence of mixed infections we compared molecular identification with results from microscopy. A consensus sequence of the forward and reverse DNA strands was obtained in Geneious R6 [45]. Next, sequences were aligned in MAFT [46], inspected and adjusted manually in Bioedit [47], and translated into amino acids, to verify that amplified copies are actually functional coding sequences. This process also allowed identifying parasite lineages shared by different species of birds, and generating an alignment of unique sequences by eliminating duplicates, but keeping track of the individual samples carrying each lineage. We used BLAST searches to compare consensus sequences to those deposited in MalAvi [48], the international database for avian haemosporidian parasites (accessed on 21.4.17; http://mbio-serv2.mbioekol.lu.se/Malavi/blast.html). It should be noted that since primers HAEMF and HAEMR2 amplify just a portion of cytb, it is possible that an identity match of 100% in BLAST actually does not correspond to the lineage stored in this database, because amplification of a larger portion of the gene could allow detecting differences between the compared sequences and therefore, identify different species or haplotypes [48]. New lineages were named following the recommendations of Bensch et. al [48] and submitted to GenBank database.

To explore the phylogenetic relationships among our sequences we created an expanded alignment, incorporating those sequences that according to MalAvi had 100–99% similarity with those amplified herein. The expanded alignment was analyzed under Bayesian and Maximum Likelihood phylogenetic approaches. Two sequences from *Leucocytozoon* (the most closely related parasite to *Plasmodium* and *Haemoproteus*) were used as out-group [49]. To build the phylogenies, we first estimated the best fit models of nucleotide substitution using jModeltest [50] based on the Akaike Information Criteria corrected for small data samples (AICc). Bayesian and Maximum likelihood analyses were then performed in CIPRES Science Gateway server [51], assigning the best model family indicated by jModeltest. In the Bayesian analysis, 2,000,000 generations were necessary to reach convergence of the runs. Convergence of the parameter values from four chains where confirmed by a standard deviation of split frequencies < 0.01. In the Maximum likelihood analysis, 10 independent runs were completed to assure consistency of likelihood scores among runs. Default values were used for other GARLI settings, as per recommendation of the developer [52]. Phylogenetic analyses had the purpose of identifying lineages according to phylogenetic signal, and not only based on sequence similarity.

### Mosquito sampling and identification

To determine vector abundance in each forest fragment, a light trap with air gate, without CO_2_ (BioQuip Products) was placed for vector capture between edge and fragment center (approximately at 150 m from the edge). The trap was mounted at a height of 1.5 m from the ground and was operative from 13:30 h of the first day to 06:30 h of the second day. At the end of the day, the mosquitos were frozen at -20°C and then transferred to 96% alcohol. Our intention was to capture biting midges (Diptera: Ceratopogonidae), vectors of subgenus *Parahaemoproteus*, and mosquitos (Diptera: Culicidae) vectors of *Plasmodium* parasites. We assumed that the probability of vector capture was directly proportional to vector abundance around the trap [53]. By using a magnifying glass, biting midges were counted and classified to family Ceratopogonidae [54] and mosquitoes to family Culicidae [55]. Also, we searched for the presence of louse flies (Diptera: Hippoboscidae), known vectors of subgenus *Haemoproteus* parasites. Since louse flies are usually found on birds´ body, we looked for their presence while birds were manipulated. Culicidae mosquitoes were classified into males and females. Biting midges were not sexed.

### Habitat characterization and environmental variables

Variables describing habitat type within each fragment were taken at 10-m intervals along a transect of 5 × 500 m from edge to center. At each point, we estimated the number of trees with a diameter at breast height (DBH) ≥10 cm and ≥50 cm, and the number of the *Cecropia* trees (a genus typical indicating disturbed forests). We also estimated canopy openness (as an index of light availability) following Browne & Karubian [56], and canopy height of the tallest tree over the center of each subunit, using a digital rangefinder. To synthesize the information of the habitat type in one factor, we performed a PCA summarizing DBH (10 and 50 cm breast height), canopy openness, canopy height, and number of *Cecropia* trees.

Fragment area was calculated by using the QGIS software using the perimeter points obtained with the GPS [57]. In the study area, areas closer to BBS have higher elevation, rainfall, and lower temperature [56], therefore, and avoid an excess of factors in the models, we decided to include only the variable elevation in the analyses. We measured elevation with a GPS. Finally, as a measure of historical habitat loss we estimated amount of tree cover around fragments from satellite images in the year 2013 (100 = complete tree cover, 0 = no tree cover) following the methods of Browne and Karubian [56].

### Relationships of forest characteristics on haemosporidian infections

Relationships between forest characteristics and haemosporidian infections were investigated by applying a binomial GLMM mixed model with a logit link function. Since all infections detected by microscopy were also confirmed by PCR, we used molecular data to determine infection status. In the models, infection status was selected as dependent variable and the following variables related to forest characteristics were included as independent variables: fragment area, forest quality (PCA), tree cover around fragments and mist-net location (edge or interior). To control for other potential factors related to infection status we also included elevation (m a.s.l.), date of bird sampling (day 1 = 1 of August), vector abundance and Shannon birds’ diversity in each fragment. We included bird species and fragment as random factors to control for the potential effect of these variables on infection status.

We used two different models to study the effect of ecological variables on infection status: one for individuals infected with *Plasmodium* and the other one for individuals infected with *Haemoproteus*. Given the low infection level detected for both parasites, we decided to perform the model-based statistical analyses with parasite lineages as pools, instead of individual lineages [13,58]. We created one pool with all *Plasmodium* lineages and the other one with all *Haemoproteus* lineages. In *Plasmodium* models, given the low abundances observed for Culicidae mosquitoes in light traps, we used presence/absence instead of mosquitos’ abundance as a covariate. In *Haemoproteus* models, *Culicoides* abundance was also included as a factor. To avoid multicollinearity among factors, a variance inflation factor (VIF) analysis was performed before running the models [59]. Model selection was performed as follows. Initially, a general model was performed including all factors (only main effects were included in the models). Then, AIC-based model selection was performed to select for the best model. A complementary analysis was performed by model averaging the bests models in a range of a Delta < 2, an accepted threshold of likely similar models [60]. Model averaging created a new model weighting for the relative importance of each term in the best models. For all models the full average model was reported and all variables were standardized before model averaging [61]. All tests were performed in R statistical software [62] with MASS [63], MuMIn [64], and Lme4 [65] packages.

### Effect of forest characteristics on vector abundance

The differences in Culicidae abundance among fragment characteristics were investigated by applying a binomial GLM model with a logit link function. Presence/absence of genus *Culex* (vector of avian *Plasmodium*) [66] was selected as dependent variable, whereas forest area, forest quality (PCA), tree cover around fragments, elevation (m a.s.l.), and date of bird sampling were selected as factors. Model selection was performed as previously. The differences in *Culicoides* abundance among fragment characteristics were investigated by means of a GLM model with a negative binomial distribution [59]. *Culicoides* abundance was selected as dependent variable and the same factors as selected in *Culex* analysis. Model selection was performed as before.

## Results

### Bird sampling and parasite identification

A total of 504 individuals belonging to 8 families and 18 species were assayed for haemosporidian infections (Table 2); the mean number of captured individuals per species was 28 ± 18.91 (mean ± SE). Microscopy inspection of smears confirmed that three bird species were infected by *Plasmodium*, and three by *Haemoproteus* parasites genus (Table 2). When present, prevalence of infection was low except for the Orange-billed Sparrow (*Arremon aurantiirostris*) and White-bearded Manakin (*Manacus manacus*), which presented elevated *Plasmodium* and *Haemoproteus* infections, respectively.

**Table 2:** Number of individuals infected by *Plasmodium* or *Haemoproteus* parasites among bird species in northwest Ecuador.

Family	Species	N	*Plas* (Micro/PCR)	Molecular lineages	*Hae* (Micro/PCR)	Molecular lineages
Bucconidae	*Malacoptila panamensis*	30	0/0		0/0	
**Emberizidae**	***Arremon aurantiirostris***	**28**	**7/17**	**ARRAUR01**^**NL**^**, ARRAUR02**^**NL**^**, BAEBIC02, DENPET03, ICTCAY01, PADOM09, PADOM11**	**3/3**	**ARRAUR03** ^**NL**^
Furnariidae	*Dendrocincla fuliginosa*	42	0/0		0/0	
**Furnariidae**	***Glyphorynchus spirurus***	**28**	**0/1**	**BAEBIC02**	**0/0**	
Furnariidae	*Sittasomus griseicapillus*	10	0/0		0/0	
Pipridae	*Lepidothrix coronata*	10	0/0		0/0	
**Pipridae**	***Manacus manacus***	**67**	**0/0**		**10/15**	**MANMAN01** ^**NL**^
**Thamnophilidae**	***Poliocrania exsul***	**47**	**0/6**	**POLEXS01**^**NL**^	**0/0**	
**Thamnophilidae**	***Myrmotherula schisticolor***	**11**	**1/1**	**ELAPAR01**	**0/0**	
**Thamnophilidae**	***Thamnophilus atrinucha***	**11**	**1/2**	**THAATR01** ^**NL**^**, THAATR02** ^**NL**^	**0/0**	
Trochilidae	*Eutoxeres aquila*	13	0/0		0/0	
Trochilidae	*Phaethornis yaruqui*	50	0/0		0/0	
**Trochilidae**	***Threnetes ruckeri***	**25**	**0/0**		**1/1**	**TROAED18**
Troglodytidae	*Microcerculus marginatus*	14	0/0		0/0	
Tyrannidae	*Leptopogon superciliaris*	10	0/0		0/0	
Tyrannidae	*Mionectes oleagineus*	61	0/0		0/0	
Tyrannidae	*Mionectes olivaceus*	36	0/0		0/0	
Tyrannidae	*Myiobius barbatus*	11	0/0		0/0	

*Abbreviations and symbols*: *N* Number of individuals analyzed, *Hae Haemoproteus* parasite, *Micro* Microscopy, *PCR* Polymerase chain reaction, *Plas Plasmodium* parasite, *NL* New Molecular lineages. In bold are species infected with either parasite.

Results of PCR detection were qualitatively similar to those of microscopy (Table 2), with 5 of 18 species infected by *Plasmodium* parasites and 3 of 18 by *Haemoproteus* parasites. Infection with *Plasmodium* parasites was positive by PCR but negative by microscopy in the wedge-billed woodcreeper (*Glyphorynchus spirurus*) and the Chestnut-backed Antbird (*Poliocrania exsul*). For *Haemoproteus* parasites, all individuals that were positive by microscopy were also positive by PCR. Corroborating the microscopy analysis, two species, Orange-billed Sparrow (*Plasmodium*) and White-bearded Manakin (*Haemoproteus*) had markedly higher infection status than did other host species. One Orange-billed Sparrow individual presented both *Plasmodium* and *Haemoproteus* infections.

In total, 11 *Plasmodium* and 3 *Haemoproteus* cytb lineages were detected. Six *Plasmodium* lineages had a 100% match to current lineages deposited in Malavi database (DENPET03, ICTCAY01, PADOM11, PADOM09, BAEBIC02, ELAPAR01), and the other five lineages (ARRAUR01, ARRAUR02, POLEXS01, THAATR01, THAATR02) were considered new lineages (Accession numbers: MH729380, MH729381, MH729384, MH729385, MH729386). With respect to *Haemoproteus*, only one lineage had a 100% match to current lineages deposited in MalAvi, (TROADE18), therefore the other two lineages (ARRAUR03, MANMAN01) were considered new (Accession numbers: MH729382, MH729383). All lineages were found infecting one species, except for BAEBIC02 (*Plasmodium*) that infected two different species belonging to different families, Orange-billed Sparrow (Emberizidae) and Wedge-billed Woodcreeper (Furnariidae). There were differences in overall infection status among bird species (*Plasmodium*: LR Chisq = 89.4, Df = 17, P < 0.001; *Haemoproteus*: LR Chisq = 49.9, Df = 17, P < 0.001).

### Cytochrome b parasite phylogeny

**Fig 2:**
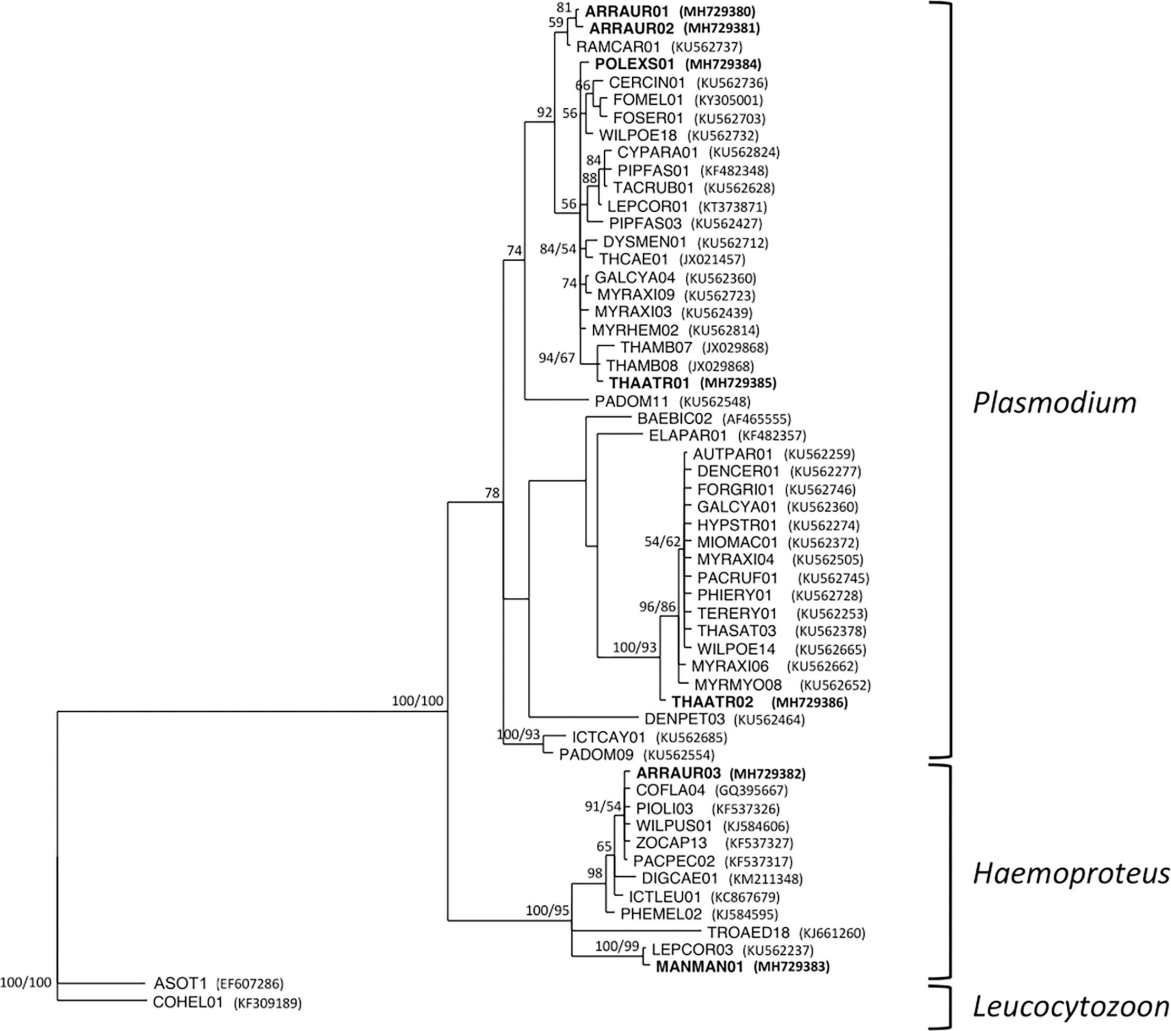
Bayesian and maximum likelihood phylogenetic trees of parasite lineages found herein in the context of similar lineages found on MalAvi. Left numbers indicate Bayes posterior probability values; right numbers indicate maximum-likelihood bootstrap; only support higher to >50% is shown.

Both Bayesian and Maximum likelihood trees showed similar sequences topology supported both by Bayesian posterior probabilities and bootstrap (Fig 2). The new lineages were closely related to other published lineages that differed on the families they infected and their distribution range (S1 Table). Lineages ARRAUR01 and ARRAUR02 (found in the Orange-billed Sparrow, family Emberizidae) were closely related to a *Plasmodium* lineage infecting birds from family Fringillidae. Lineage THAATR01 (found in the Slaty Antwren, family Thamnophilidae), was closely related to lineages also infecting birds from the same family. POLEXS01 lineage (found in the Chestnut-backed Antbird, family Thamnophilidae) was closely related to a big group of *Plasmodium* sequences, the same group where THAATR01 lineage was found. This group included lineages found in birds from a diverse group of families. Lineage THAATR02 appeared in a different cluster than THAATR01 and was closely related to lineages infecting also a broad spectrum of families. With respect to *Haemoproteus* parasites the new lineage ARRAUR03 (found in the Orange-billed Sparrow) was closely related to lineages of different families. Outside this group, MANMAN01 lineage (found in the White-bearded Manakin) was closely related to a lineage found in the same family Pipridae, and also in Thamnophilidae and Tyrannidae.

### Forest characteristics and bird parasite infection status

**Fig 3:**
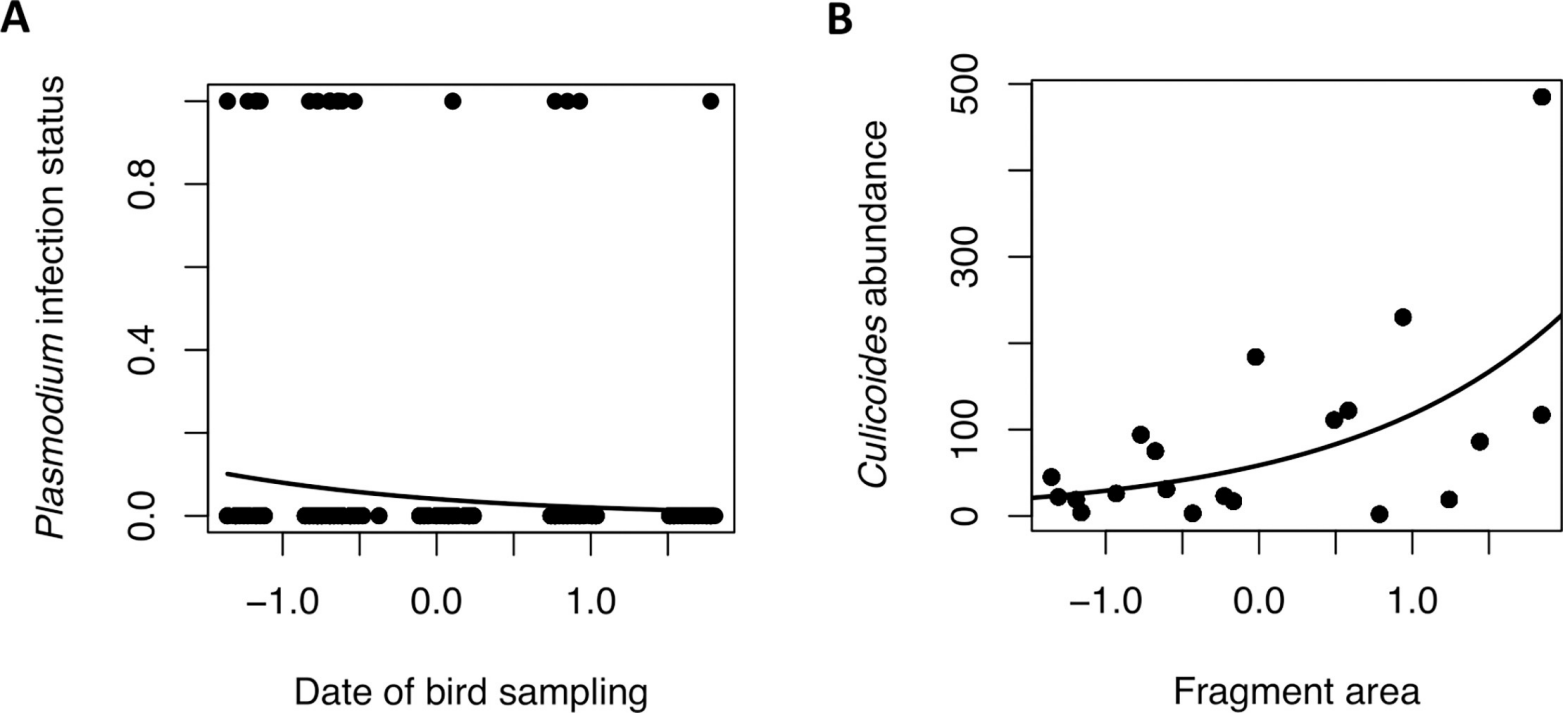
A. Scatter plot of the relationship between date of bird sampling and *Plasmodium* infection status (0 = Non-infected, 1 = Infected). B. Scatter plot of the relationship between fragment area and *Culicoides* abundance. Fitted model values were obtained with predict function in R. N = 504 individuals.

We interpret our first principal component (S2 Table) as an index of how pristine the forest structure in each fragment was, with forest fragment quality being positively associated with canopy height, number of both 10 and 50-DBH trees, and negatively associated to number of *Cecropia* trees and canopy openness. For *Plasmodium*, AIC-based model selection resulted in a best model where date of bird sampling was the only factor related to infection status of birds (Table 3). Individuals sampled at the end of the dry season had lower *Plasmodium* infection status than individuals sampled at the beginning of the of dry season (Fig 3A), with no evidence of an effect from forest characteristics *per se* on bird infection status. However, R-squared values indicated that most of the explained variability were due to the random factors. Model averaging of the best models confirmed also date as the only one factor associated to infection (S3 Table). With respect to *Haemoproteus* infections, AIC-based model selection resulted in a best model that included tree cover around fragments, however this term was not statistically significant (Table 3). Model averaging did not find tree cover around fragments related to *Haemoproteus* infection status (S3 Table).

**Table 3:** AIC-based best model selection of the effect of forest characteristics on *Plasmodium* and *Haemoproteus* sp. infection status and vectors’ presence and abundance.

		Estimate	Std. Error	Z-value	P-value	R^2^ marginal	R^2^ conditional
***Plasmodium* infection status**							
	Intercept	-7.23	2.56	-2.86	0.004 *	0.06	0.851
	Date	-1.21	0.4	-2.97	0.002 *		
***Haemoproteus* infection status**							
	Intercept	-4.99	0.94	-5.27	<0.0001 *	0.09	0.564
	Cover	-0.83	0.44	-1.84	0.06		
***Culex* presence**							
	Intercept	1.87	1.19	1.57	0.116	0.12	
	Elev	-0.004	0.003	-1.37	0.168		
***Culicoides* abundance**							
	Intercept	3.11	0.41	7.57	<0.0001 *	0.28	
	Area	0.06	0.02	3.16	0.001 *		

*Abbreviations*: *Area* forest fragment area (in ha), *Cover* tree cover around fragments, *Elev* meters above sea level (m a.s.l.).

For both parasites, we explored if the overall result from models could be caused by the general low prevalence observed in the study. In this case, we repeated the analyses only for the two species with the highest prevalence of infection, the Orange-billed Sparrow and the White-bearded Manakin, but uncovered qualitatively similar results. In the case of the Orange-billed Sparrow (infected with *Plasmodium*), date of bird sampling was associated with infection status (Model averaging, Date: P = 0.03) and for White-bearded Manakin (infected with *Haemoproteus*), no factor had any effect on infection status (P > 0.1).

### Forest characteristics and vector abundance

Light traps captured a total of 1715 biting midges of the family Ceratopogonidae, and 74 mosquitoes of family Culicidae (Table 1). Biting midges belonged to the genus *Culicoides*, a vector of *Haemoproteus*. The mean number of *Culicoides* per fragment/sample was 85.7 ± 113.2 (mean ± SD) with a range of 2–485. Culicidae mosquitoes belonged to genus *Culex* (vector of *Plasmodium* parasite). The mean number of *Culex* per fragment/sample was 3.5 ± 7.1 (mean ± SD), with a range of 0–29. Mean abundance of *Culex* was 46.9% males and 53.1% females. No blood was observed in any mosquito’s abdomen. No louse fly was observed on any individual.

Forest characteristics were not associated to either presence/absence of *Culex*. Model selection resulted in a best model with elevation as the only retained term, however it was not statistically significant (Table 3). Model averaging of the best models retained elevation and tree cover around fragments as factors, however no term was significant (S4 Table). On the other hand, forest area was positively related to abundance of *Culicoides* (Fig 3B). Best model and model averaging found fragment area positively related to *Culicoides* abundance (Table 3 and S4 Table). To disentangle the relationship between fragment area and *Culicoides* abundance, we explored if larger fragments were sampled at the beginning of the study, however, fragment size was not related to date of capture (Pearson test: r = -0.09, p = 0.68). Finally, we explored if *Culex* presence and *Culicoides* abundance decreased throughout the study, but there was no variation for any kind of vector (*Culex*, Pearson test: r = 0.09, df = 18, p = 0.67; *Culicoides*, Pearson test: r = 0.09, p = 0.48).

## Discussion

In this study, we investigated how characteristics of forest fragments are associated with infection by avian malaria (*Plasmodium*) and the related haemosporidia parasite (*Haemoproteus*), in rainforest dwelling birds in northwest Ecuador. We found no associations between fragment characteristics (e.g., area, habitat quality) or landscape attributes (e.g., elevation, tree cover around fragments) and infection status of birds. However, we observed differences in infection status among bird species and a effect from seasonality, in which *Plasmodium* infection rates declined as sampling extended from the end of the rainy season thru the dry season. On the one hand, our results are in accordance with other studies where no association was observed between habitat loss and avian haemosporidians [21]. For example, in a Brazilian Atlantic forest, although forest fragment size was related to changes in host populations and vectors, no effect was found on avian blood parasites [67]. However, also in the same region, birds captured in larger areas were more infected than those captured in smaller ones [68]. At the same time, other studies have found evidence of an effect from habitat loss on avian malaria. In Cameroon, for example, birds from undisturbed forests had a higher prevalence of *Plasmodium* infections than did birds from disturbed ones [13]. A trend supported in another study in the same region, where undisturbed habitats positively correlated with higher prevalence of *Plasmodium*, *Haemoproteus*, and *Leucocytozoon* parasites [69]. Also, in northern Australia, higher prevalence in undisturbed tropical forests has been found [10]. Therefore, more research is still needed to better understand the effect of habitat loss on avian haemosporidian infections, especially in tropical forests.

Differences on parasite infections may be due to a variety of other factors, including the phylogeny of birds [32,70,71], avian community composition [67,72], bird species [14], food availability, altitude or woodland edge [30,73], microhabitat [74] or landscape characteristics [75]. As observed in other studies, we observed marked differences in infection status depending on bird species, a factor that explained most of the infection variability in our models. Overall, a low parasite infection was found in all but two species, the Orange-billed Sparrow and the White-bearded Manakin. Low prevalence of infection of haemosporidian parasites has been observed in other studies carried out in the Neotropics [32]. Factors like host-parasite assemblage or host immunocompetence has been proposed to explain for the absence of parasites or low prevalence of infection [32,76]. Thus, it is possible that the overall low infection observed in our study is masking the patterns of infection in the avian community. In a study carried out throughout Ecuador, prevalence of infection varied among different sites in a range from 7 to 60% [33] and higher elevations and lower temperatures were the most important explanatory factors. Harrigan et al. [33] included samples from BBS, the large undisturbed forest adjacent to our study area, and mean prevalence from BBS was 30% in a set of different species, potentially indicating a higher prevalence in BBS compared to our fragments (4%). Contrarily to our study Harrigan et al.[33] did not detect any *Plasmodium* lineage in BBS, whereas we detected three lineages of this genus in the surrounding forest fragments. The presence of this parasite in the fragments but absent in BBS might be caused by habitat loss outside BBS. *Plasmodium* parasites behave in general as generalist [31], that is, are able to infect more than one host species, therefore this condition could confer an advantage in fragmented landscapes. Alternatively, the discrepancy between studies could be explained by small overall sample sizes in both cases, associated with relatively low sampling effort given low rates of infection, or by differences in month sample collection by our study and in Harrigan et al (2014), which is not reported.

Another factor that may explain the lack of association between characteristics of the fragments and avian malaria infections is birds’ mobility among fragments. If a bird has the potential to move easily among fragments, it is then virtually impossible to link an individual’s infection to an original fragment. This mobility could bias infection status in relation to the place of sampling. We controlled for movements of birds among fragments by bird banding, but we had no recaptures. Also, we estimated tree cover around fragments, a measurement of suitable habitat that allows movements of birds among fragments. For example in Barbados, *Haemoproteus* parasites were absent from regions scarce in vegetation [77]; therefore tree cover could be an explanatory variable of current surrounding vegetation, but no such associations were found in our study. Also we investigated edge-effects to control for differences in forest dependency behavior on parasite infections, but they had no relevancy in the models (see also Laurance et al. [10]). In Japan, it was found that species richness and abundance was greater in larger forests, whereas small forest had more abundance of generalist birds [78]. We captured both dependent and non-dependent forest birds, therefore, we cannot rule out the possibility of movement of birds among them.

One factor that can help to disentangle the associations between forest characteristics and haemosporidian infections is the inclusion of the presence and abundance of vectors. Haemosporidians are transmitted by mosquitoes that need suitable environmental conditions; therefore, vector presence or abundance may explain the chances to become infected. In our study, there was a reduction in *Plasmodium* infections, and this could be explained by a reduction in *Culex* presence, yet no reduction was observed during the season. Therefore, the decreasing of *Plasmodium* infections was not explained by a variation in the presence of vectors. However, we did not use CO_2_ traps, and this likely affected the performance of the light traps attracting Culicidae mosquitoes and our results could be affected by the low sampling effort for *Culex* mosquitoes.

On the other hand, we investigated variations in infection status related to rainfall and we observed a reduction in *Plasmodium* infections throughout the study. This reduction, although significant was subtle and independent of elevation. Therefore, we found support from an overall effect of seasonality but not related to local environmental conditions. A reduction of infections has been observed in tropical forests due to a decrease of precipitation [35]. In temperate regions, haemosporidian parasites increase their abundance in the blood stream to increase chances of transmission, and decrease its abundance during unfavorable conditions, for example, when vector activity decrease or is inexistent [79]. In tropical regions seasonality exists due to differential rainfall throughout the year, then, it is possible that precipitation affected vectors activity. However, an increase of prevalence during the dry season have been also reported, coinciding with birds breeding [80], therefore the effect from seasonality on infection status could exhibit different scenarios. Finally, although *Culex* is a vector of avian malaria parasites, only some species has been involved in its transmission. A more detailed study and identification of Culex’ species in the region would be necessary to identify the actual vectors of avian *Plasmodium*. In the case of *Haemoproteus*, we found a positive relationship between fragment area and *Culicoides* abundance; however, vector abundance was not related to an increase on *Haemoproteus* infections. The lack of association between *Culicoides* abundance and higher prevalence could be due to our experimental design or caused by other factors, for example if larger forests were sampled at the beginning of the study, but there was no association between fragment size and date of capture sampling.

In conclusion, the observation of an absence of an effect from habitat loss on haemosporidian infections in this study does not conclusively imply there is no such effect. Rather, the low prevalence observed could be the result of a drastic fragmentation in the region or the fact that most of our sampling was conducted during the dry season. Because parasite load is closely associated with the feeding behavior of mosquito vectors [81,82] the inclusion of parasite load instead of parasite prevalence as the proxy for parasite infection may shed new light on understanding the relationship between habitat loss and parasite infection in the area. Finally, the finding of an association between seasonality and avian malaria infections highlights the importance of this factor in future studies, particularly when working in the tropics.

## Supporting information

S1 Table Geographic areas and families of other molecular lineages. (DOCX)

S2 Table Principal component analysis (PCA) of habitat type. (DOCX)

S3 Table Model averaging of the associations of habitat loss and ecological variables on infection status. (DOCX)

S4 Table Model averaging of the associations among forest characteristics and vector abundance. (DOCX)
