# Supplementary material for: Patterns of avian haemosporidian infections vary with time, but not habitat, in a fragmented Neotropical landscape
Source: PLoS One. 2018 Oct 31;13(10):e0206493. doi: 10.1371/journal.pone.0206493 (PMC6209335; doi:10.1371/journal.pone.0206493)
Supplement: S1 Table — (DOCX) [file pone.0206493.s001.docx]

**Supporting information**

S1 Table. Geographic areas and families of other molecular lineages from MalAvi database closely related to the new lineages found in this study.
