# Supplementary material for: Patterns of avian haemosporidian infections vary with time, but not habitat, in a fragmented Neotropical landscape
Source: PLoS One. 2018 Oct 31;13(10):e0206493. doi: 10.1371/journal.pone.0206493 (PMC6209335; doi:10.1371/journal.pone.0206493)
Supplement: S2 Table — (DOCX) [file pone.0206493.s002.docx]

S2 Table. Principal component analysis (PCA) of habitat type among forest patches sampled in northwest Ecuador. *Abbreviations*: *DBH 10* number of trees with a diameter at breast height ≥10, *DBH 50* number of trees with a diameter at breast height ≥50, *Cecropia* number of the *Cecropia* trees.
