# Supplementary material for: Patterns of avian haemosporidian infections vary with time, but not habitat, in a fragmented Neotropical landscape
Source: PLoS One. 2018 Oct 31;13(10):e0206493. doi: 10.1371/journal.pone.0206493 (PMC6209335; doi:10.1371/journal.pone.0206493)
Supplement: S3 Table — (DOCX) [file pone.0206493.s003.docx]

**Supporting information**

S3 Table. Model averaging of the associations of habitat loss and ecological variables on infection status. *Abbreviations*: *Area* forest fragment area (in ha), *Cover* tree cover around fragments (%), *Date* date of birds sampling, *Elev* meters above sea level, *PCA* habitat type principal components analysis.
