# Supplementary material for: Patterns of avian haemosporidian infections vary with time, but not habitat, in a fragmented Neotropical landscape
Source: PLoS One. 2018 Oct 31;13(10):e0206493. doi: 10.1371/journal.pone.0206493 (PMC6209335; doi:10.1371/journal.pone.0206493)
Supplement: S4 Table — (DOCX) [file pone.0206493.s004.docx]

S4 Table. Model averaging of the associations among forest characteristics and vector abundance. *Abbreviations*: *Area* forest fragment area (in ha), *Cover* tree cover around fragments (%), *Date* date of birds sampling, *Elev* meters above sea level, *PCA* habitat type principal components analysis.

|  |  | Estimate | Std. Error | Z-value | P-value |
| --- | --- | --- | --- | --- | --- |
| *Culex* *sp.* presence/absence |  |  |  |  |  |
|  | Intercept | 2.25 | 5.12 | 0.41 | 0.676 |
|  | Elev | -0.001 | 0.002 | 0.54 | 0.586 |
|  | Cover | -0.01 | 0.05 | 0.24 | 0.808 |
|  |  |  |  |  |  |
| *Culicoides sp.* abundace |  |  |  |  |  |
|  | Intercept | 2.21 | 3.67 | 0.58 | 0.559 |
|  | Area | 0.06 | 0.02 | 2.14 | 0.032 * |
|  | PCA | 0.006 | 0.05 | 0.1 | 0.913 |
|  | Cover | 0.01 | 0.04 | 0.24 | 0.806 |
|  | Date | -0.00006 | 0.001 | 0.03 | 0.975 |
|  | Elev | 0.00001 | 0.0004 | 0.03 | 0.976 |
